# Label-free pathology by spectrally sliced femtosecond stimulated Raman scattering (SRS) microscopy

**DOI:** 10.1371/journal.pone.0178750

**Published:** 2017-05-31

**Authors:** Andrew Francis, Kyla Berry, Yikai Chen, Benjamin Figueroa, Dan Fu

**Affiliations:** Department of Chemistry, University of Washington, Seattle, Washington, United States of America; Tufts University, UNITED STATES

## Abstract

Optical “virtual biopsy” is an attractive way to improve disease diagnosis and surgical guidance. Many optical microscopy techniques have been developed to provide diagnostic information without the need for tissue sectioning or staining. Among these techniques, label-free chemical imaging is the most desirable. Recently, it has been shown that narrowband, picosecond stimulated Raman scattering (SRS) can achieve comparable morphological contrast to hematoxylin and eosin staining (H&E staining), the ‘gold standard’ of pathology. However, to translate the technique from the bench to the bedside, optimal laser sources and parameters have yet to be identified. Here we describe an improvement to the narrowband SRS microscopy techniques for label-free tissue imaging. Through spectral slicing of broadband, femtosecond pulses, we are able to maintain the same protein/lipid contrast as narrowband SRS while achieving a higher signal-to-noise ratio (SNR). Our method draws upon the benefits of femtosecond pulses (e.g. higher peak power) while preserving those of picosecond pulses (e.g. adequate spectral resolution). We demonstrate this achievement through protein/lipid signal and contrast quantification of mouse brain tissue as a function of bandwidth, and comparison with numerical simulations. Further method validation is provided through imaging of additional mouse tissues: liver, kidney, and skin.

## Introduction

Since its discovery over a hundred years ago, hematoxylin and eosin staining (H&E staining) has long served as the ‘gold standard’ of histopathology for achieving strong visual contrast between various cellular features [1]. This contrast is achieved via chemical labelling of specific functional groups present throughout the tissue giving rise to spatial-chemical distribution maps of the tissue. Exploiting key chemical differences between healthy and diseased cells, pathologists can use cellular morphology to not only diagnose the disease but the severity of the condition as well from such staining [2]. While H&E staining is not likely to be replaced by any current technologies, the requirement of tissue excision, sectioning, and staining severely limits its use in an intraoperative setting. Quick and accurate diagnosis is crucial for effective surgical treatment of a variety of cancers including brain, breast, prostate, etc., in which any residual tumor will increase the risk of cancer relapse. Therefore, a real-time assessment of histopathological features of either excised tumor tissue or the surgical cavity may significantly improve the outcome of the patient [3].

Several competing optical techniques have been developed to address the challenge of intraoperative tissue assessment. One powerful tool is optical coherence tomography (OCT), a low-coherence interferometric technique that images tissue structure at high resolution based on variations in refractive index [4–6]. *In vivo* OCT was initially developed for ophthalmology, but has been rapidly extended to epidermal, cervical, esophageal, and gastrointestinal tissues [7]. Most recently, it has been used for detection of brain tumor infiltration [8]. OCT has a relatively large penetration depth of 1–3 mm, compared to other optical microscopy techniques. However, it suffers from a lack of chemical specificity and thus is unable to provide detailed cellular morphology.

In order to gain chemical contrast, many groups have worked to use autofluorescence and fluorescent staining coupled with confocal or multiphoton microscopy to visualize cellular structures. Although more chemically specific than OCT, both confocal and multiphoton fluorescence are still limited in contrasts: only a few native *in vivo* species are known to autofluoresce, most notably collagen. For tissues that lack strong autofluorescence, an exogenous agent is added to promote strong fluorescent contrast. The two main staining methods are 5-aminolevulinic acid (5-ALA)-induced protoporphyrin IX and nuclear staining, commonly achieved using acridine orange. With nuclear staining, both confocal and multiphoton microscopies have been demonstrated to provide cellular contrasts similar to H&E that enables rapid *ex vivo* assessment of tumor margins. [9,10]. The use of fluorescent dyes does not preclude fluorescence microscopy from *in vivo* imaging, as demonstrated by 5-ALA stained, fluorescence-guided resection surgeries [11–14], but does present additional avenues of intrasample and intersample discrepancies. Dye-based methods suffer from drawbacks including heterogeneous delivery, nonspecific staining, and FDA approval limitations [15].

Stimulated Raman scattering (SRS) microscopy is an emerging analytical, label-free technique that has demonstrated exceptional *ex vivo* [16–20] and *in vivo* [19, 20] tissue imaging capabilities. Compared with fluorescence, it provides specific chemical contrasts without the need for staining. Raman scattering is the inelastic collision between an incident photon and a molecule. The transfer of energy excites a vibrational mode unique to that molecule, making Raman scattering highly qualified to study inhomogeneous systems such as biological tissues. The advent of coherent Raman scattering microscopy addressed the major limitation of applying Raman scattering to tissue imaging—the data acquisition speed. Coherent Raman scattering, including both coherent anti-Stokes Raman scattering (CARS) and SRS, improves the feeble spontaneous Raman scattering effect by a few orders of magnitude, allowing rapid image acquisition at a speed up to video-rate [21]. Traditionally, SRS is achieved using narrowband, picosecond pulses tuned to a particular Raman transition that has a resonance frequency Ω equal to the frequency difference between the pump and the Stokes laser ω_P_ — ω_S_ [22]. The most commonly used laser system is neodymium based oscillator and a synchronously pumped optical parametric oscillator. The spectral resolution of SRS imaging is determined by the bandwidths of the picosecond lasers, which are typically less than 10 cm^-1^. In order to open SRS up to multi-band excitation, many groups began experimenting with broadband, femtosecond pulses. Multi-band excitation has been reported via frequency tuning [20], multiplexing [23, 24] and spectral focusing [25]. These studies paved the way for broadband, femtosecond SRS as an alternative to the traditional narrowband, picosecond SRS.

Recently, the viability of narrowband, picosecond SRS imaging of brain tumor margins with both frozen and fresh tissue as an alternative to H&E staining has been demonstrated [17, 18, 26]. In the work reported by Ji et al. [17, 18], two-color SRS was adopted to acquire additional spectroscopic information, allowing for differentiation of various biochemical and morphological features. For biological samples, the two Raman transitions used are typically near 2845 and 2930 cm^-1^ for lipids and proteins, respectively. In the study conducted by Lu et al. [26], three-color SRS was employed, where the third channel probed corresponds to hemoglobin. While the work reported by Ji et al. and Lu et al. demonstrates the powerful capabilities of their respective systems, the lasers they used have 7 ps long pulses, which suffer from low peak intensity and require high laser power for imaging. Since SRS signal scales with peak intensity, theory posits that spectrally broader, temporally shorter pulses provide higher signal-to-noise ratio (SNR) at the same optical power than narrowband, picosecond pulses. One notable study, conducted by Freudiger et al., detailed the improvement in SNR using spectrally broader, temporally shorter 2 ps pulses through a fiber-based SRS set up [27]. However, fiber-based set ups are limited by their own shortcomings, including high frequency laser noise and difficulties generating sufficiently high power compared to free-space lasers. Regardless, the work done by Freudiger et al. demonstrated that temporally shortened pulses produce higher SNR. Such a system has been recently used by Orringer for intraoperative histology of unprocessed surgical specimen [28].

One important question that remains unsolved in two-color SRS based histopathology is what the optimal laser pulses for imaging are. In one study, a near 12-fold increase in SNR of lipids SRS was observed when the traditional narrowband, picosecond pulses were replaced with broadband, femtosecond SRS [29]. However, there is no systematic study of the influence of pulse duration on SNR. More importantly, spectral broadening associated with shorter pulses results in loss of spectral resolution. It is questionable that in two-color SRS imaging whether the same cell morphological contrasts can be obtained with broadened pulses. In this manuscript, we present two-color SRS tissue imaging using spectrally sliced broadband, femtosecond pulses to systematically investigate the influence of excitation laser bandwidth on SNR and image contrast. After spectral unmixing, our SRS images can provide visual protein/lipid contrast comparable to narrowband, picosecond SRS, and thus, analogous to H&E staining. Importantly, we find that a linear increase in SNR is achieved with pulse bandwidth increase up to 40 cm^-1^, while almost identical two-color contrasts are retained compared to narrowband pulses. The results agree with our numerical simulations. This finding has wide implications for future development of SRS imaging systems for intraoperative tumor margin assessment and early tumor diagnosis.

## Methodology

**Fig 1:**
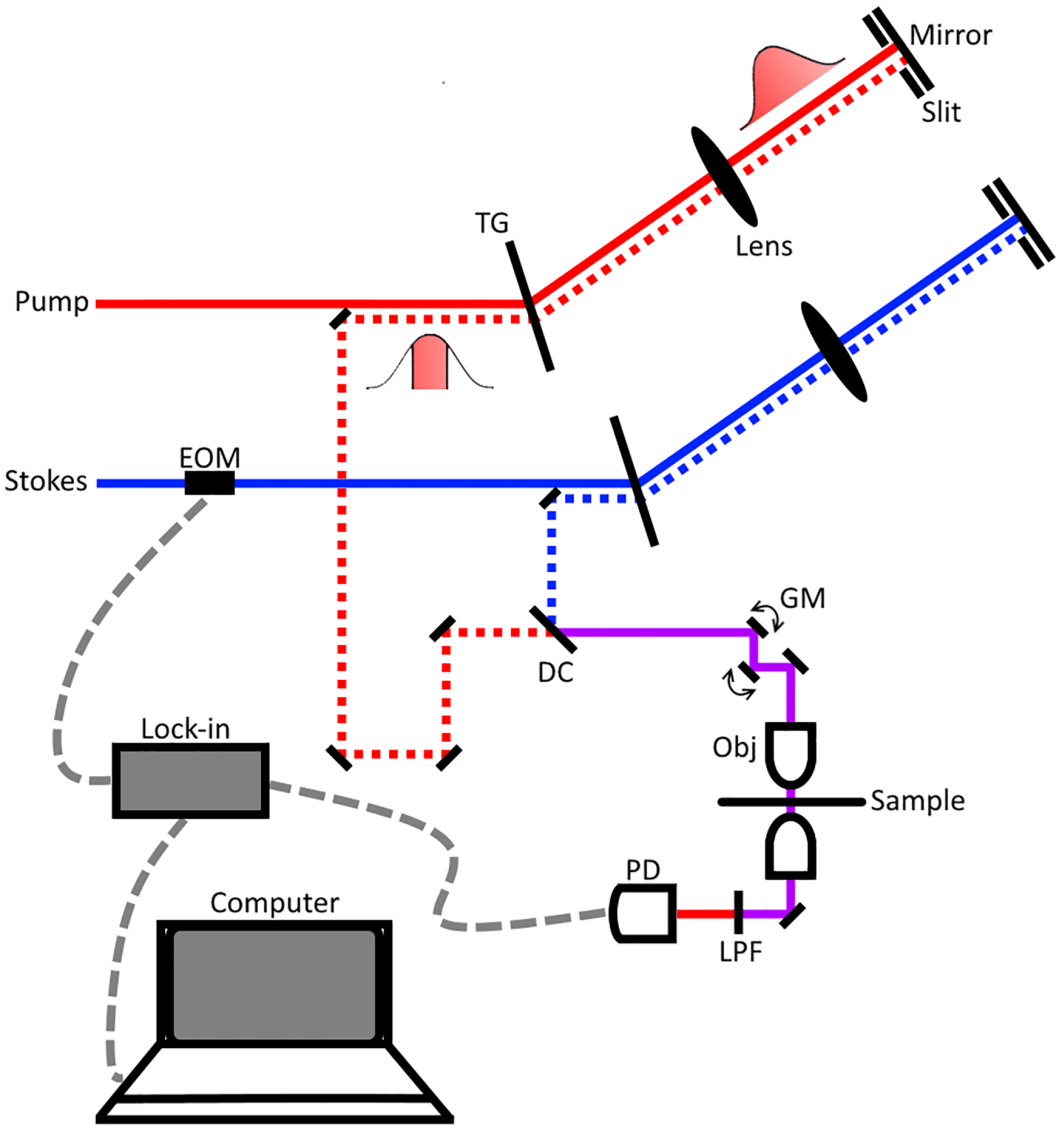
Experimental spectral slicing set up. The Stokes laser is modulated by an electro-optical modulator (EOM) at 10 MHz. Pulses are sliced via spectral dispersion through a transmission grating (TG) and a controllable slit placed near the focal point. After slicing, pulses are spatially and temporally synchronized at a dichroic mirror (DC) and directed onto a pair of galvanomirrors (GM). The beams are then sent through the 40x objective, sample, and condenser. Finally, the Stokes beam is filtered out using a low pass filter (LPF) before the beams terminate at the photodiode (PD). Dotted red and blue lines represent an offset in the beam height and the dashed grey lines detail the electrical connections between computer, PD, and lock-in amplifier.

Our experimental set up used is shown in Fig 1. The spectral slicing process begins with a broadband femtosecond dual beam system (Insight DS+ from Spectra Physics) with an 80 MHz repetition rate. The two outputs are: a fixed output of 1040 nm with an average power of 800 mW and bandwidth of 60 cm^-1^ and a variable output from 680–1300 nm with an average power of 1.2 W and bandwidth of 150 cm^-1^. Immediately following emission from the laser, the Stokes beam is modulated at 10 MHz using an electro-optical modulator (EOM). Next, both pulses, pump and Stokes, are spectrally dispersed by a transmission grating, physically sliced with a slit, and returned through the grating at a lower height than incidence to recollimate. The sliced pulses are then combined at a dichroic mirror and overlapped temporally using a delay line in the pump arm. The resulting beam is sent into a home-built laser scanning microscope. A 40X Nikon water immersion objective (Nikon 40X, NA = 1.25) is used to focus the beams onto to the tissue sample. At the focus, the Stokes beam has an average power of 35 mW and the pump has an average power of 40 mW. With a combined power of ~75 mW at the focus, we are imaging with similar optical power as recently reported for *in vivo* imaging of mouse brain vasculature [30], and 2-3-fold less optical power than other ultrafast *ex vivo* multiphoton imaging studies [17, 18, 31]. Moreover, Galli et al observed little to no photodamage of *ex vivo* mouse brain tissue under similar excitation parameters [32]. After passing through the condenser, the Stokes beam is filtered out and the pump reaches a silicon photodiode. Stimulated Raman loss (SRL) signal is detected with a home-built lock-in amplifier [21]. The layout was inspired by work reported by Zhang et al. using a 4-f system to achieve angle-to-wavelength pulse shaping [33]. Ours differs by adopting a “folded” 4-f system that uses the grating to recollimate the sliced pulses.

Both the pump and Stokes pulses are narrowed using moveable slits that allow for control of bandwidth and central wavelength. The center wavelength and bandwidth of each pulse after slicing were verified using a home-built Michelson interferometer-based spectrometer.

Brain, liver, kidney, and skin tissues were harvested from recently sacrificed mouse carcasses, which were provided by UW Animal Use Training Services (AUTS) according to IACUC protocol 3388–03. The mice (laboratory mice, *mus musculus*) were euthanized by isoflurane or CO_2_ overdose and cervical dislocation. The brain was exposed and extracted by removing the scalp and top of the skull. The kidney and liver were collected by making a Y-incision to expose the intestines then clipping the connective tissues. Skin tissues were excised using scissors after hair was removed with Nair. After excision, bulk tissues were placed in phosphate-buffered saline (PBS). Thin sections of tissue were prepared by razor blade sectioning and then placed on a glass slide with additional PBS. To prepare the sample for imaging, a glass coverslip was placed on top to sandwich the tissue specimen between the glass slide and the glass coverslip using double-sided tape as a spacer.

## Results and discussion

**Fig 2:**
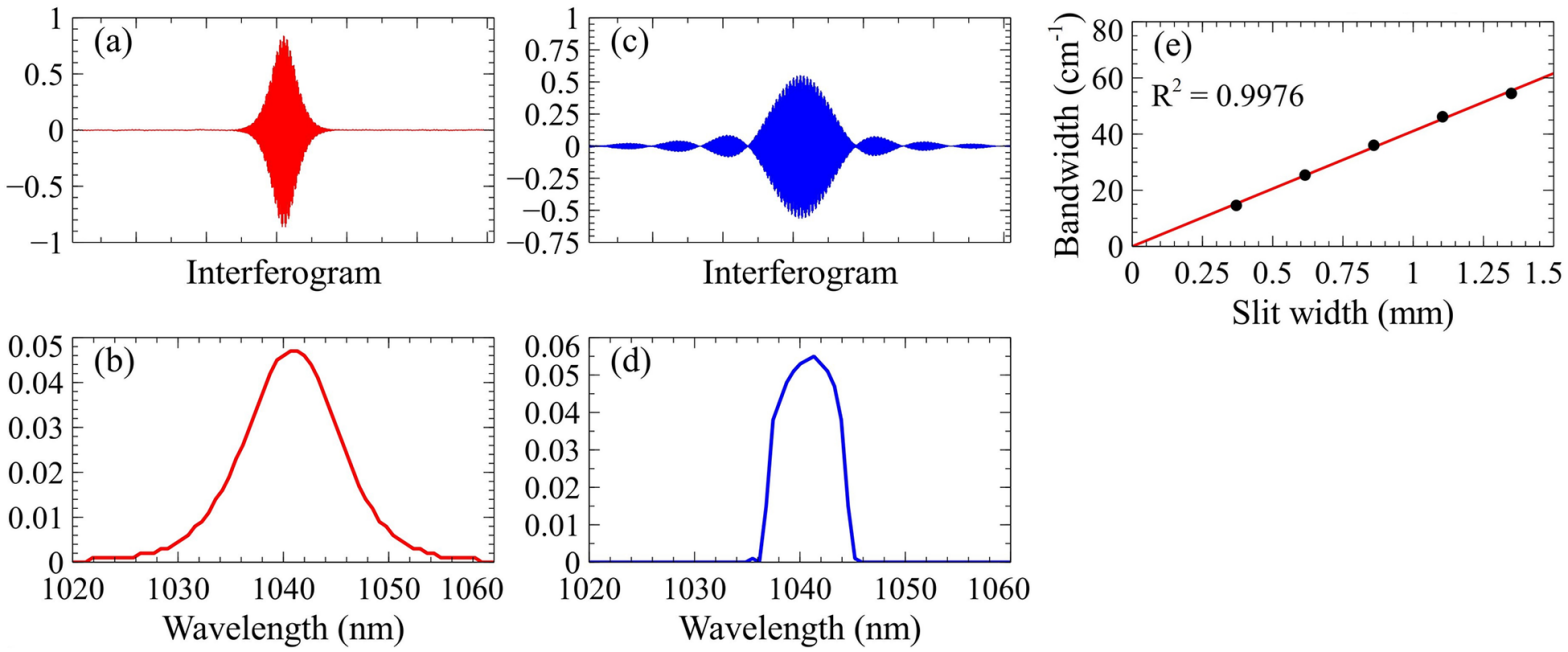
Effects of slicing on Stokes pulse. (a) Unsliced interferogram of Stokes pulse using a Michelson interferometer-based spectrometer. (b) Unsliced Stokes frequency profile via Fourier transform (FT) of unsliced interferogram. (c) Sliced interferogram of Stokes pulse. (d) Sliced Stokes frequency profile via FT of sliced interferogram. (e) Linearity of bandwidth as a function of slit width.

We first examined the results of spectral slicing using the aforementioned spectrometer. Fig 2 illustrates the effects of slicing on the Stokes pulse and provides the linear calibration of the slit width. The collected interferograms of the unsliced Stokes pulses are collected and the calculated spectra via Fourier transform (FT) are displayed in Fig 2(a) and 2(b), respectively. As evident by these two figures, the pulses produced by the laser are Gaussian-like in distribution. Spectral slicing induces a convolution of the laser pulse (Gaussian) and the slit (rectangle), shown in Fig 2(c) and 2(d). After slicing, the interferogram more closely represents a sinc function (sin(x)/x), rather than Gaussian. We define the spectral bandwidth as the full width at half maximum (FWHM) in the frequency domain relative to the central wavelength. Fig 2(e) demonstrates the linearity of sliced spectral bandwidth as a function of slit width.

The Fourier transform relationship between frequency and time dictates that as a pulse narrows spectrally, it broadens temporally. Therefore, the pulse duration of an unsliced pulse is narrower than that of a sliced pulse. However, the cost of stronger peak intensity is reduced SRS spectral resolution of the selected Raman transition. In S1 Fig, the difference between using narrower (10 cm^-1^) and broader (60 cm^-1^) bandwidths is illustrated by highlighting the integration interval difference. Using 10 cm^-1^ pulses, the 2855 cm^-1^ channel is predominately lipid signal and the 2935 cm^-1^ channel is more protein than lipid. When the bandwidth is increased to 60 cm^-1^ each channel becomes less specific due to higher contributions from the competing chemical (e.g. lipid signal in the 2935 cm^-1^ channel).

**Fig 3:**
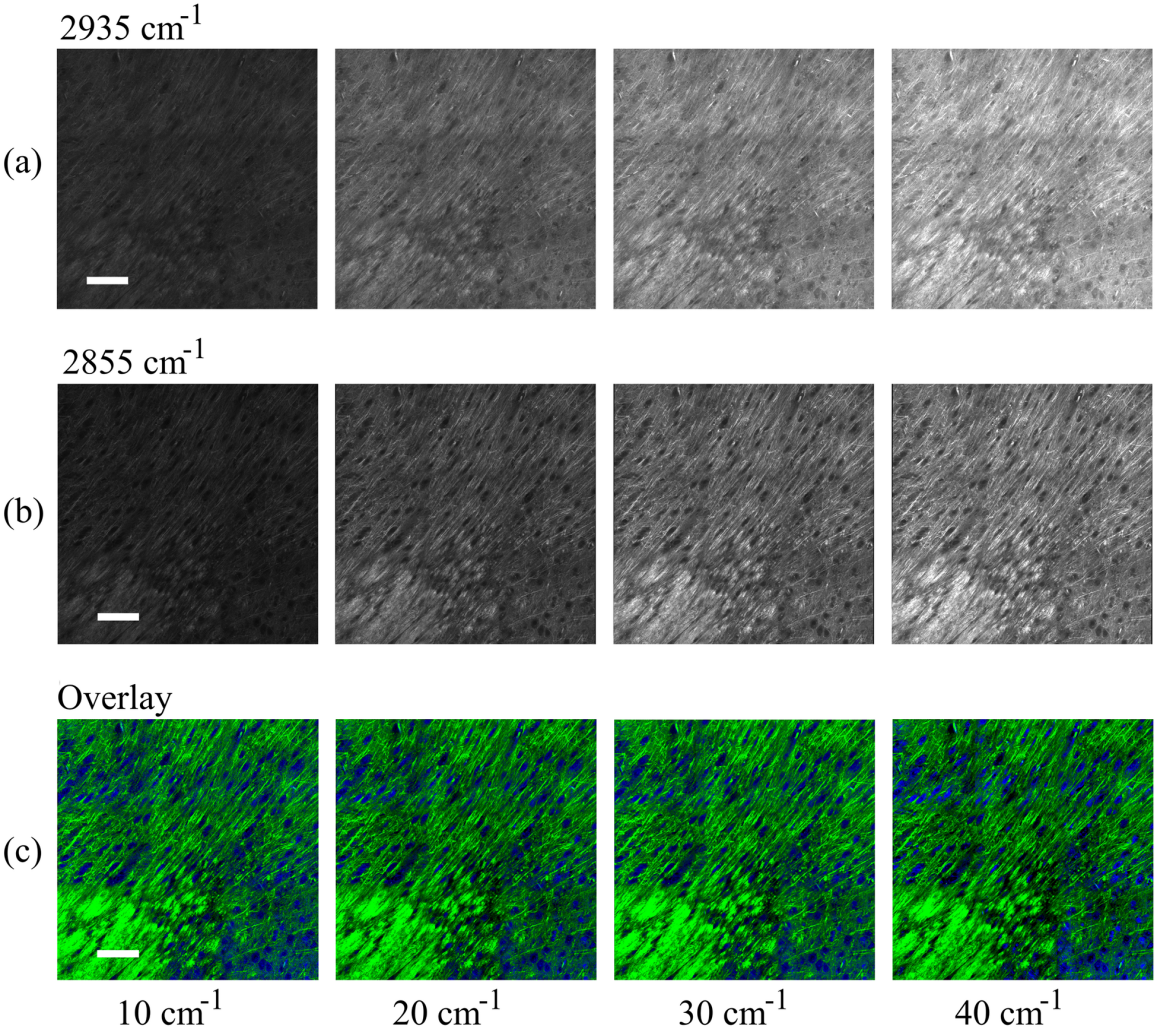
SRS imaging of mouse brain tissue. Imaging at (a) 2935 cm^-1^ (proteins) with bandwidths of 10 cm^-1^, 20 cm^-1^, 30 cm^-1^, and 40 cm^-1^ from left to right and (b) 2855 cm^-1^ (lipids) with bandwidths of 10 cm^-1^, 20 cm^-1^, 30 cm^-1^, and 40 cm^-1^ from left to right. As expected, the mean intensity increases as the bandwidth broadens. After unmixing (subtracting the lipids out of the protein channel) the images are color coded and merged; the proteins are colored blue and the lipids, green. Composite tissue images (c) show only slight differences between 10, 20, 30, and 40 cm^-1^ from left to right. Scale bar: 100 μm.

To study this phenomenon, we imaged cross-sections of freshly harvested mouse brain at multiple bandwidths. Fig 3 demonstrates the increase in SNR associated with broadening the pulses from 10 to 40 cm^-1^ at constant excitation power. The images shown have been field normalized to mitigate the field effects caused by the imaging and stitching process. All of the images shown are on the same brightness and contrast scale allowing for a noticeable increase in signal intensity to be clearly observed. Next, the contribution of protein and lipids are unmixed using the same procedures as published by Ji et al. [18]. This process is achieved by removing the lipid contribution from the protein channel via subtraction. To quantify the contribution of lipids in each channel, we measure the response of oleic acid (representative of cellular lipids) at each bandwidth for both channels. Knowing the contribution at a particular bandwidth for each channel allows us to appropriately scale the protein channel (2935 cm^-1^) and subtract out the lipid channel (2855 cm^-1^). Fig 3 provides the unmixed protein (a) and lipid (b) images and the overlay images (c) of mouse brain tissue; the protein channel is shown in blue and the lipid channel, green. The images have been shown at similar brightness and contrast scales to more easily compare the images between bandwidths. Features rich in protein, such as nuclei, appear blue, while features rich in lipids, such as myelin sheaths, appear green. From Fig 3, no apparent changes in contrast are easily observable.

**Fig 4:**
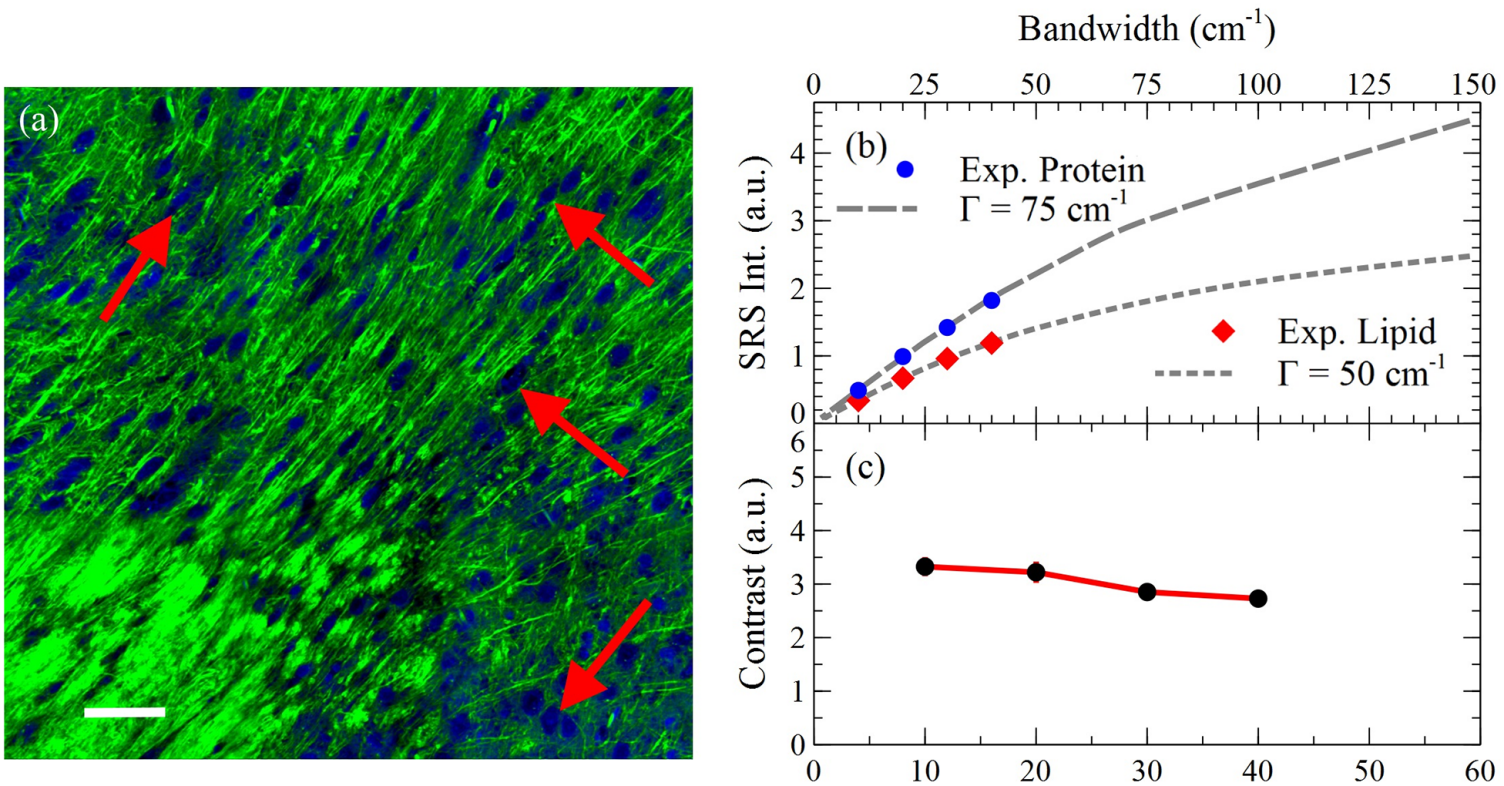
Signal and contrast dependence on bandwidth. (a) SRS imaging of mouse brain tissue 30 cm^-1^ pulses. Total signal for the (b) lipid and protein channels as a function of bandwidth are also shown to illustrate the increase in signal as bandwidth increases. The dashed and dotted gray lines are theoretical curves at 75 and 50 cm^-1^, respectively. (c) Contrast at four areas was averaged and plotted as a function of bandwidth. The image shown represents the four areas used and is the overlay at 30 cm^-1^ from Fig 3 for reference. Scale bar: 200 μm.

To quantify the variation between the images, the contrast between the two channels for several features was calculated and averaged. We define protein/lipid contrast as the ratio between protein rich nuclei and lipid rich cytoplasm. The results of this analysis and features used are shown in Fig 4.

In Fig 4 three curves are shown demonstrating (b) lipid and protein signal intensity and (c) protein/lipid contrast as a function of bandwidth. Fig 4(b) shows the agreement of experimental findings with our numerical simulation (Matlab 2016, Mathworks) detailing the relationship between SRS signal intensity and bandwidth/pulse duration using a previously derived formula [34]. Our simulation demonstrates that this relationship is dependent upon the bandwidth of the excitation pulses and the bandwidth of the Raman transition, Γ. More specifically, when excitation pulses are narrower than the Raman transition, the curve is linear; in the opposite case, the curve flattens out and plateaus. For cellular lipids and proteins, Γ is large, which we believe is responsible for the linearity of Fig 4(b). By approximating the Raman transition of proteins and lipids to be 75 and 50 cm^-1^, respectively, we see that the experimental findings correlate well with the theoretical model. The linear regime of both curves is important because it suggests that optimized excitation conditions exist: inside the linear regime, SNR increases linearly with laser bandwidth; outside the linear regime, the risk of photodamage quickly increases with only marginal increase of SRS signal. Fig 4(c) relates the contrast between the channels to the excitation bandwidth. The trend shows a modest decrease in protein/lipid contrast with increasing bandwidths, with the most substantial being the increase from 20 to 30 cm^-1^. We believe that this is due to other chemical species present in the sample that are not taken into account in spectral unmixing [16]. In this study, we only consider the two most prevalent species, proteins and lipids, and neglect contributions from others, most notably water and nucleic acids. Water has a very broad band from 2900 to 3600 cm^-1^ with maxima around 3200 and 3400 cm^-1^, and nucleic acids span 2850 to 3100 cm^-1^ with a maximum near 2955 cm^-1^. Therefore, these species will predominantly contribute to the protein channel at 2935 cm^-1^, with increasing contribution at larger bandwidth. These contributions confound spectral unmixing and diminish image contrast.

**Fig 5:**
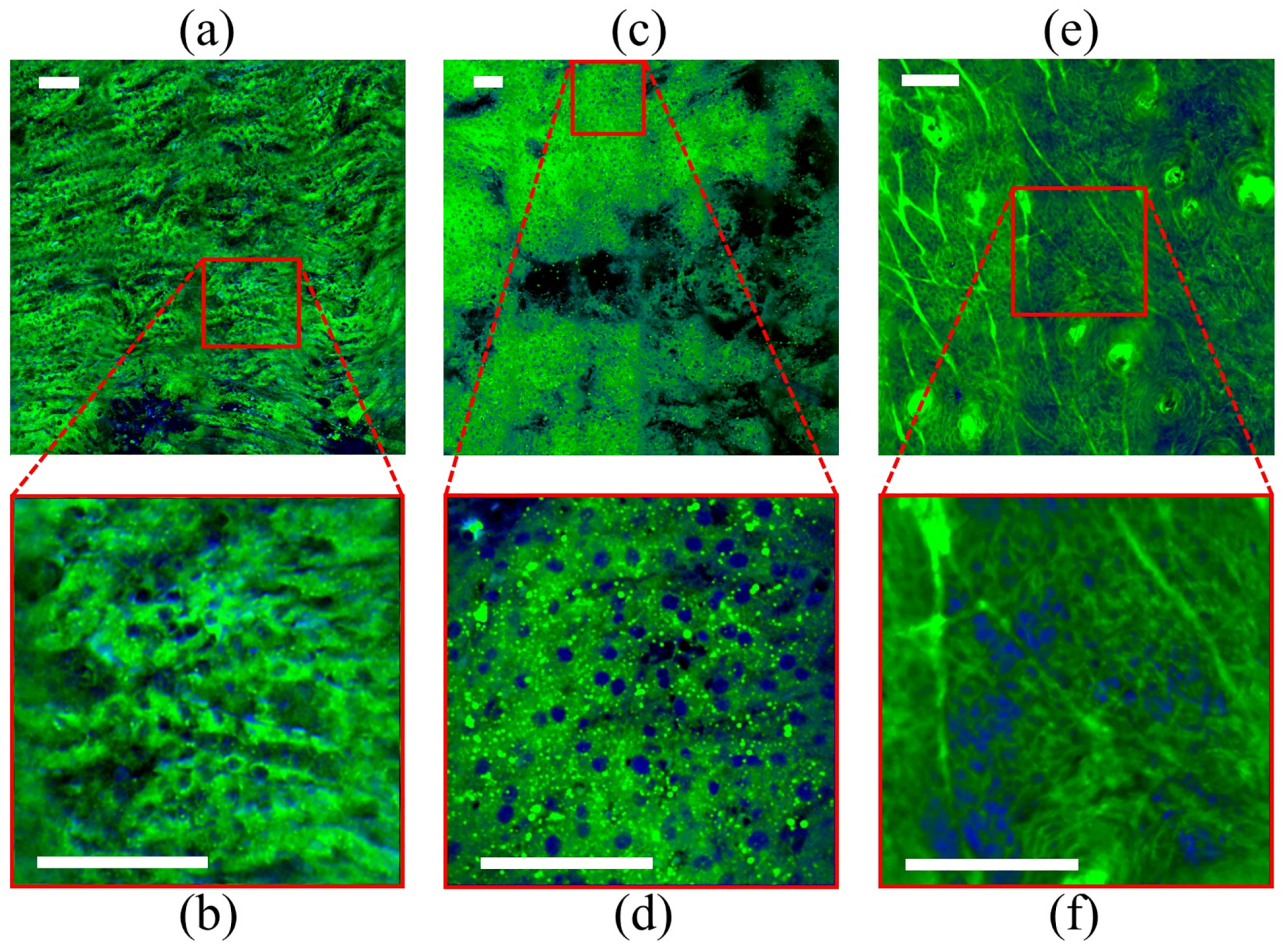
Broadband, femtosecond SRS imaging of mouse tissue using 40 cm^-1^ pulses. The tissues shown are (a, b) kidney, (c, d) liver, and (e, f) skin. For each tissue, the above image is a larger field of view (FOV) and the below image is a smaller FOV indicated by the red outline. After unmixing, the images are color coded and merged; the proteins are colored blue and the lipids, green. Scale bars: 100 μm.

To verify our system’s capabilities on a variety of biochemical compositions, we also imaged three other mouse tissues: liver, kidney, and skin. Fig 5 provides the post-processing overlay images of kidney (a, b), liver (c, d), and skin (e, f) using 40 cm^-1^ excitation pulses. Fig 5(a), 5(c) and 5(e) all show a large field of view (FOV) and Fig 5(b), 5(d) and 5(f) gives a smaller FOV at the specified location. From these images, it is evident that broadband pulses are equally effective at providing cellular morphological contrast across a wide range of chemical compositions.

**Fig 6:**
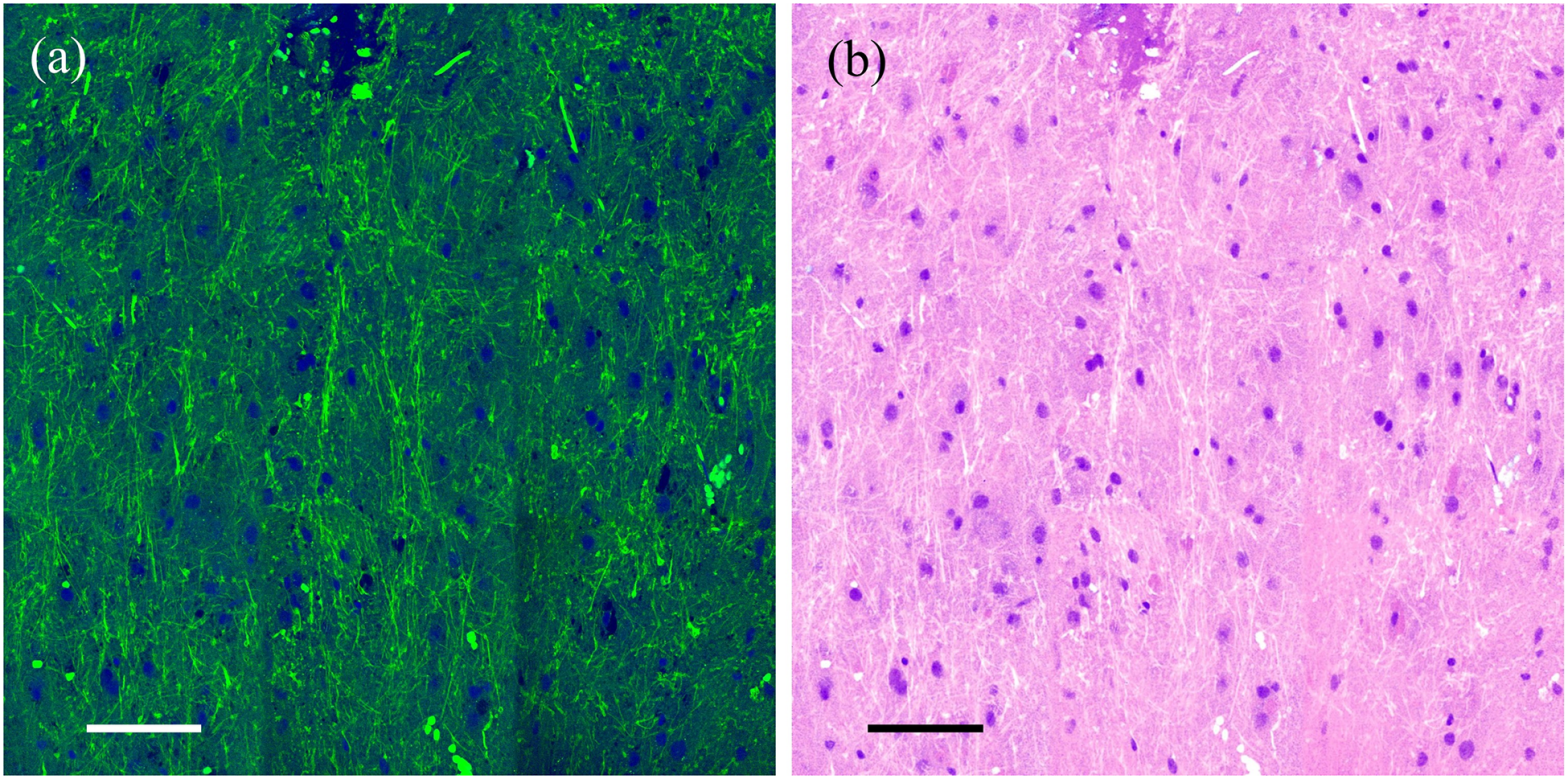
Simultaneous two-channel SRS imaging of mouse brain tissue using 30 cm^-1^ pulses. The mouse brain image shown was collected using dual-phase lock-in detection [32]. (a) Shows the proteins as blue and the lipids, green, while (b) provides a pseudo-H&E color scheme. Scale bars: 100 μm.

Spectral slicing is a robust method of shaping pulses to very controllable bandwidths/pulse durations; however, a notable disadvantage is the loss of power associated with the physical slicing of the laser beams. One alternative method to pulse shaping is spectral focusing, wherein the excitation pulses are chirped through highly dispersive glass rods [25,35]. In spectral focusing, the Raman transition to be probed is chosen by the temporal delay between the pump and Stokes beams. We used dual-phase lock-in detection that has recently demonstrated by He to perform simultaneous two-color SRS imaging [35]. In our experiment, much shorter pulses were used for imaging. In Fig 6, we provide the results of simultaneous two-channel SRS imaging of freshly excised mouse brain tissue using 30 cm^-1^ excitation pulses with a combined power of 80 mW at the focus. The two transitions shown are 2850 cm^-1^ for lipid and 2945 cm^-1^ for protein excitation. These transitions and bandwidth differ from those used above due to technical differences between spectral slicing and two-channel SRS. Fig 6(a) provides the color scheme used above, while Fig 6(b) remaps the color scheme to a pseudo-H&E color scale [36].

## Conclusion

In summary, the use of spectrally sliced broadband, femtosecond SRS provides similar protein/lipid contrast to narrowband, picosecond SRS after channel unmixing. The main benefit of using broader pulses is increased SNR which is crucial for improving imaging speeds and the limit of detection of SRS. To this extent, we have characterized the relationship between protein/lipid contrast and spectral bandwidth and shown that contrast only decrease slightly by going from 10 cm^-1^ to 40 cm^-1^. Moreover, we have demonstrated the optimization of excitation parameters for proteins and lipids within the linear regime between signal intensity and excitation laser bandwidth. We further demonstrated that simultaneous two-color SRS imaging can be achieved with spectral focusing instead of spectral slicing, which more efficiently uses the laser power. The optimal bandwidth for excitation depends on both contrast and photodamage. In previous demonstration of two-color SRS based histopathology [17, 18, 26, 28], 7 ps and 2 ps pulses were used for SRS excitation. It was shown that 2 ps pulses provide better SNR than 7 ps pulses given the same laser power. For transform-limited pulses, 2 ps pulses correspond to ~5–10 cm^-1^ laser bandwidth. Our experiment demonstrates that a further 4-fold increase in SNR can be gained by using even shorter pulses without significant loss of morphological contrasts. Although bandwidths larger than 40 cm^-1^ may still provide contrasts without much degradation, the gain in SNR rapidly decreases due to the limit in Raman transition width of proteins and lipids according to our numerical simulation. Moreover, the photodamage due to high peak power may be of concern at very large laser bandwidth. Our experimental results are promising as a benchmark in two-color SRS based pathology applications. Yet, more work needs to be done regarding broadband, femtosecond SRS. Although we have demonstrated the viability of our system on imaging a wide variety of tissue with varying lipid content, for a more complex tissue environment that contains substantially different biochemical composition or tissue inhomogeneity, our system still needs to be tested. Future work will focus on comparing broadband two-color SRS imaging of human tumor tissues to H&E staining to validate its diagnostic capability.

The benefits demonstrated in this manuscript are essential for the integration of SRS into an intraoperative/guided surgery setting. First, as we, and other groups, have demonstrated, the switch from traditional picosecond excitation lasers to femtosecond lasers is accompanied by significant SNR increase. Theoretically, an order of magnitude increase in SNR is expected for 40 cm^-1^ broadband pulses compared to the most common 7 ps laser system given the same incidence power. With higher SNR, less optical power can be used to achieve the same quality of images and imaging speed which mitigates the risk of potential photodamage. Another benefit of broadband, femtosecond SRS is the choice of laser source. In translating from bench to the bedside, fiber laser is poised to play an essential role in clinical imaging. Due to high nonlinearity associated with femtosecond pulses, it is often simpler to construct femtosecond lasers compared to picosecond lasers. As a result, more femtosecond lasers are available on the market. Finally, broadband, femtosecond pulses allows simultaneous two-color SRS imaging due to the proximity of the protein and lipid peaks, as demonstrated in this manuscript. Simultaneous two-color SRS offers quicker data acquisition by removing the time required to switch wavelengths, which is necessary for narrowband, picosecond SRS. Furthermore, as demonstrated herein by the results of two-color unmixing, protein/lipid visual contrast is not significantly affected by the bandwidth of the pulses used, within the range of widths detailed. Thus, a carefully optimized broadband, femtosecond system could provide sufficient protein/lipid contrast at significantly higher imaging speed than current narrowband, picosecond two-color SRS.

## Supporting information

S1 Fig Dependence of signal amplitude and contrast on bandwidth. Normalized spontaneous Raman spectra of bovine serum albumin (BSA) and oleic acid are shown as representative of proteins and lipids, respectively. Shown is the difference in excitation of the protein channel (2935 cm^-1^) and the lipid channel (2855 cm^-1^) with (a) 10 cm^-1^ pulses and (b) 60 cm^-1^ pulses.
(TIF)
