# Supplementary figures and images for: Label-free pathology by spectrally sliced femtosecond stimulated Raman scattering (SRS) microscopy
Source: PLoS One. 2017 May 31;12(5):e0178750. doi: 10.1371/journal.pone.0178750 (PMC5451135; doi:10.1371/journal.pone.0178750)

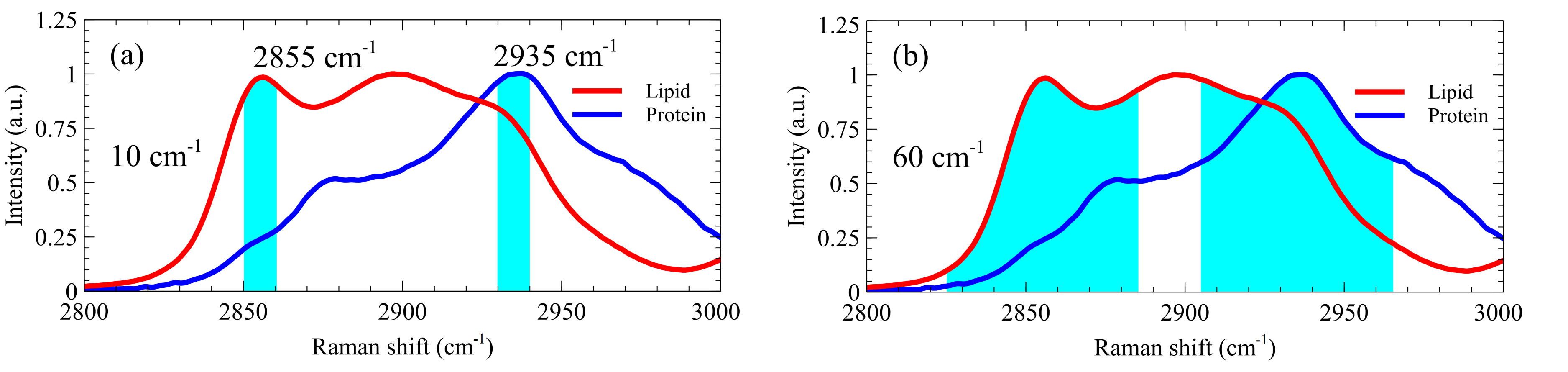

Supplement: S1 Fig — Normalized spontaneous Raman spectra of bovine serum albumin (BSA) and oleic acid are shown as representative of proteins and lipids, respectively. Shown is the difference in excitation of the protein channel (2935 cm-1) and the lipid channel (2855 cm-1) with (a) 10 cm-1 pulses and (b) 60 cm-1 pulses. (TIF) [file pone.0178750.s001.tif]
